# Evaluation of diphtheria surveillance system in Kaduna State, Nigeria, July 2023 – December 2023

**DOI:** 10.4102/jphia.v16i1.1379

**Published:** 2025-08-27

**Authors:** Uwaifiokun J. Okhuarobo, Samuel A. Owoicho, Jeremiah Daikwo, Isiaq H. Shehu, Emmanuel Omomoh, Mfon-obong P. Ibara, Abiola O. Oshunniyi, Oladipo O. Ogunbode, Fatima Saleh

**Affiliations:** 1 Nigeria Field Epidemiology and Laboratory Training Programme, Abuja, Nigeria; 2 Veterinary Public Health and Preventive Medicine, Faculty of Veterinary Medicine, University of Abuja, Abuja, Nigeria; 3 Federal Ministry of Agriculture and Food Security, Abuja, Nigeria; 4 Kaduna State Ministry of Health, Kaduna, Nigeria; 5 Nigeria Centre for Disease Control and Prevention, Abuja, Nigeria; 6 Africa Centres for Disease Control and Prevention, Addis Ababa, Ethiopia; 7 Department of Community Health, University of Uyo, Uyo, Nigeria; 8 Clinton Health Access Initiative, Abuja, Nigeria

**Keywords:** data accuracy, diphtheria, surveys and questionnaires, disease outbreaks, health facilities, Nigeria

## Abstract

**Background:**

The re-emergence of the diphtheria outbreak in Nigeria raises concern about the surveillance system’s capability to detect, prepare for and respond to outbreaks.

**Aim:**

To evaluate the usefulness and attributes of the diphtheria surveillance system in Kaduna State, Nigeria.

**Setting:**

Kaduna State, northwest Nigeria.

**Methods:**

An observational study, using a mixed-method approach, was adopted. It comprised a survey, a record review of the 2023 outbreak and key informant interviews. A pre-tested semi-structured self-administered questionnaire and an interview guide, adapted from the Centres for Disease Control and Prevention (CDC) (2001) guidelines on surveillance evaluation, were utilised. Three stakeholders were interviewed, 21 surveillance officers were surveyed, and eight surveillance attributes were assessed.

**Results:**

The outbreak data had 67% (*n* = 382/573) missing values. Eighty-two per cent (*n* = 432/525) of cases had an investigation initiated within 48 hours. All 21 (100%) respondents reported ease in filling in forms and a willingness to continue participating in the system, which could correctly identify cases. Twelve (57%) of the 21 respondents stated that private health facilities submitted their reports. Of the 12 respondents who reported modifications in the system, six (50%) stated < 1 month for implementation. Nineteen (90%) of the 21 respondents reported inadequate resources, while six (67%) of the nine respondents who reported receiving stipends indicated these were provided by partner organisations.

**Conclusion:**

The system was acceptable to stakeholders, useful in detecting outbreaks, simple in data collection, flexible in accommodating changes and sensitive in identifying cases. However, resource constraints pose a threat to its stability. We recommend providing adequate resources, improving data quality and reporting from private health facilities.

**Contribution:**

The study underscores areas for improvement in the diphtheria surveillance system, highlighting the potential for targeted interventions to overhaul the system.

## Introduction

Public health surveillance is essential for the prompt identification and response to public health threats and outbreaks.^1,2^ The diphtheria surveillance system aims at monitoring diphtheria burden, defining transmission patterns, and identifying the outbreaks promptly to avert spread.^3^

Diphtheria is reported to cause death in 5% to 10% of cases, especially among children under 15 years of age,^4^ with huge economic implications.^5^ In recent times, several nations in Africa have reported a re-emergence of outbreaks, with Nigeria accounting for the majority of the cases.^6^ Since 2017, pentavalent-1 vaccination coverage has been on a continuous steady decline in Nigeria,^5^ falling below the 90% target recommended by the World Health Organization (WHO).^7^ The re-emergence of the outbreak in Nigeria started in May 2023, and as of October 2023, Kaduna State was reported to be one of the six worst-hit states, contributing to over 95% of cases and more than 600 deaths.^8^ The Nigeria Centre for Disease Control and Prevention (NCDC) reported that, as of 31 July 2023, Kaduna State ranked as one of the top three states with suspected cases,^9^ and as of 05 October 2023, the Kaduna State Ministry of Health reported that 60% of Local Government Areas had recorded at least a confirmed case.^10^ The outbreak necessitated the deployment of two separate groups of National Rapid Response Teams (NRRTs) from NCDC to support outbreak response activities.

The re-emergence of diphtheria outbreak in Nigeria raises several public health concerns, including issues bordering on the capacity of the surveillance system to promptly detect, prepare and respond to the outbreaks. The WHO Vaccine-Preventable Diseases Surveillance Standards^3^ provide a framework for evaluating a diphtheria surveillance system. These standards closely correspond with Nigeria’s National Technical Guidelines for Integrated Disease Surveillance and Response (IDSR).^11^ The United States (US) Centers for Disease Control and Prevention’s (CDCs) updated guidelines for evaluating public health surveillance systems^12^ further strengthen this framework by providing a structured approach for assessment of the system quality as it allows to identify the areas for improvement of the system.^13^

Furthermore, the WHO recommends evaluating the surveillance system at least once a year^3^ to determine its efficacy, pinpoint areas that require enhancement, and guarantee that it remains relevant and functional in identifying and managing outbreaks. Therefore, considering the 2023 outbreak of the disease, an evaluation of the diphtheria case-based surveillance system in Kaduna State is necessary. We, thus, aimed to evaluate the usefulness and attributes of the diphtheria surveillance system in Kaduna State, Nigeria.

## Research methods and design

### Study design

A mixed-method approach was adopted. The quantitative component involved a survey of Local Government Area Disease Surveillance and Notification Officers (DSNOs) and a review of the 2023 outbreak data. The qualitative component involved the key informant interviews (KIIs) among major stakeholders from the Kaduna State Ministry of Health and NCDC.

### Setting

Kaduna State is in the northwest geopolitical zone of Nigeria. It is divided into three senatorial districts and has 23 Local Government Area.^14^ It has a projected population of 9 032 200 as of 2022,^15^ and 40% of the population are less than 15 years old.^16^

### Study population and sampling strategy

Only Local Government Area DSNOs who were available during the evaluation period were surveyed. A total of 21 (91%) of 23 Local Government Area DSNOs participated in the survey. The key informant interviews (KIIs) involved three stakeholders, namely the State Epidemiologist, the State DSNO and the NCDC State Surveillance Officer. Key informants were selected purposively based on their expertise and experience in the diphtheria surveillance system and their leadership role in the state’s health system.

### Case definitions

The case definitions were adapted from IDSR^11^ as well as the NCDC diphtheria situation report.^17,18^ A suspected case was defined as any person in Kaduna State with upper respiratory tract infection, in addition to pharyngitis, nasopharyngitis, tonsillitis or laryngitis and an adherent pseudo-membrane.^18^ A confirmed case was defined as anyone residing in Kaduna State with any of the following: (1) laboratory confirmation: ‘a person with *Corynebacterium spp.* isolated by culture and positive for toxin production, regardless of symptoms’^17^; (2) epidemiological linkage: ‘a person that meets the definition of a suspected case and is linked epidemiologically to a laboratory-confirmed case’^17^; (3) clinical compatibility: ‘a person that meets the definition of a suspected case and lacks both a confirmatory laboratory test result and epidemiologic linkage to a laboratory-confirmed case’.^17^

### Operation of diphtheria surveillance system

**FIGURE 1:**
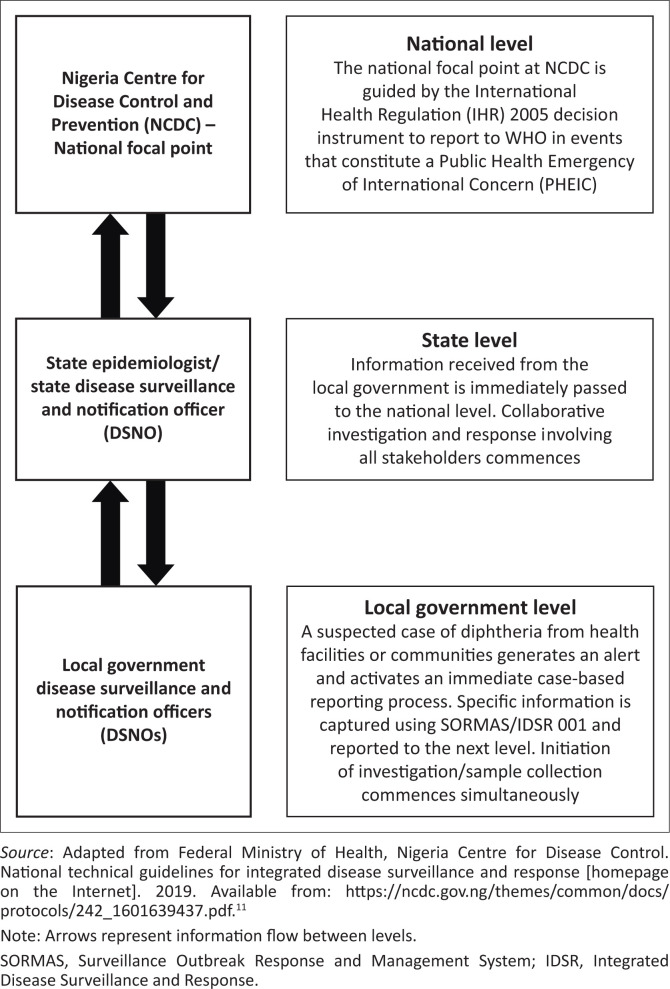
Diphtheria surveillance flowchart in Kaduna State, Nigeria.

Diphtheria is an immediately case-based notifiable disease in Nigeria and the surveillance in Kaduna State aligns with the IDSR system.^11^ It involves both passive and active surveillance components. All cases are expected to be reported immediately (or within 24 h) to the Local Government Area DSNOs by community informants, health workers and surveillance focal persons across the state. The Local Government Area DSNO is then expected to capture all case data and immediately report to the State Epidemiologist and the State DSNO using the fastest means available. They are also expected to simultaneously fill out both the electronic IDSR tools (Surveillance Outbreak Response and Management System – SORMAS) and the paper-based IDSR Form 001. All samples collected from suspected cases are sent to the National Reference Laboratory, Abuja, for confirmation. Results are line-listed in Excel format and sent to the state epidemiology unit. A flow chart of the diphtheria surveillance system in Kaduna State, Nigeria, is shown in Figure 1. It illustrates the different levels and designated responsibilities within the surveillance system.^11^

### Data collection

A pre-tested semi-structured self-administered questionnaire and an interview guide adapted from the CDC (2001) guidelines on surveillance evaluation^12^ were used for the survey and the KIIs, respectively. The domains included socio-demographic features, usefulness and attributes of the surveillance system. Data abstraction covered the 2023 outbreak period from July 2023 to December 2023, and data collected included epidemiological and laboratory data using an abstraction tool adapted from the national guidelines for IDSR.^11^ Evaluation of the surveillance system took place in February 2024.

### Method of assessment of usefulness and attributes of the system

The attributes evaluated include simplicity, flexibility, data quality, acceptability, representativeness, timeliness and completeness, stability and sensitivity (Online Appendix 1). Data quality was assessed by using a scale of 1–5 (1 = Poor, 2 = Fair, 3 = Good, 4 = Very good and 5 = Excellent). The completeness of reporting was assessed based on reporting focal sites in the state. For timeliness of case investigation, the date laboratory sample was collected was taken as the date investigation was initiated and all those who visited the health facility were taken as the number of suspected cases. The results of the evaluation of timeliness and completeness were described in comparison with the surveillance standards.^3^

### Data analysis

Categorical variables were reported as frequencies and proportions, while continuous variables were reported using appropriate measures of central tendency. Microsoft Excel^®^ 365 and Epi info 7 were utilised in analysing quantitative data from the survey and abstracted data from the 2023 outbreak line-list. Results were presented in tables and charts. A thematic analysis of the qualitative data was performed iteratively.

### Reporting framework

The Standards for Quality Improvement Reporting Excellence (SQuIRE) checklist was adapted for the reporting of this evaluation as it allows describing system-level projects for healthcare improvement^19^ (Online Appendix 2). Modification was done to the checklist to align with the CDC Updated Guidelines for Evaluating Public Health Surveillance System.^12^

### Ethical considerations

Evaluating the diphtheria surveillance system in Kaduna State involved several ethical considerations, including informed consent from stakeholders and confidentiality of collected data. An application for full ethical approval was made to the Kaduna State Ministry of Health, Kaduna State, Nigeria, and ethics consent was received on 17 January 2024. The ethics approval number is MOH/ADM/744/VOL.1/111046, NHREC/17/03/2018. Informed consent was requested and obtained from survey participants (Disease Surveillance and Notification Officers) and key informants (stakeholders) before data collection.

## Results

Twenty-one DSNOs participated in the survey, and three key informants were interviewed. The median age of respondents in the survey was 48 years (42–52 years). Seventeen (81%) of the 21 respondents were males. Twenty-one (100%) survey respondents had tertiary education level and were married. Twelve (57%) of the 21 respondents were community health extension workers, and the mean number of years of experience as a DSNO was 5 years (+4 years). The three key informants were all males, and the mean years of work experience was 3 years (+1 year). The emerging themes were centred on system performance and recommendations for improvement.

### Usefulness of the system

All (100%) survey respondents reported that the diphtheria surveillance system was useful. In addition, 20 (95%) of the 21 respondents reported that data collected at the Local Government Area level were analysed and used for decision-making at both the Local Government Area and State levels. All (100%) respondents in the survey also agreed that they get regular feedback from the State level. The key informants interviewed unanimously responded that the system is ‘data-driven, and the data informs public health actions and resource management’.

**FIGURE 2:**
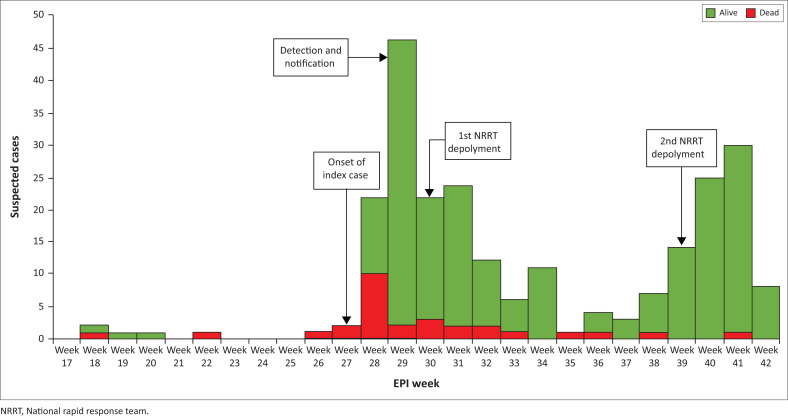
Epidemic curve of confirmed cases of diphtheria in Kaduna State as of week 42, 2023.

The 2023 outbreak line-list showed that the index case was detected and reported in epidemiological (epi) week 29, although the earliest death from the outbreak was as early as week 18 (Figure 2).

### System attributes and performance

#### Simplicity

Eleven (52%) of the 21 survey respondents reported using specific data capturing tools, which was also corroborated by the key informants. All (100%) respondents in the survey reported that the forms were easy to fill in and that a diphtheria case is easy to identify based on the case definition. The median time for filling in a Case Investigation Form was 15 min (10–30 min). A key informant, however, reported that all the variables might be ‘necessary, but too many, which may result in incomplete filling of data forms, especially during the outbreaks’.

#### Flexibility

Twelve (57%) of the 21 respondents reported that the system has had some modifications. Out of this, 6 (50%) reported that it took less than a month for the modifications to be included in the system. Nine (75%) of the 12 respondents reported that human and financial resources were used to implement these changes. A key informant reported the modifications to include ‘the review of the IDSR technical guidelines as well as introduction of SORMAS for reporting immediately notifiable diseases’.

#### Data quality

**FIGURE 3:**
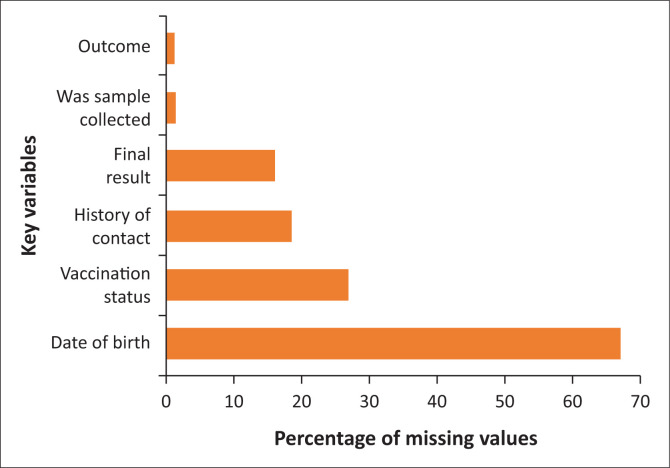
Proportion of missing values across key variables in the 2023 outbreak line-list in Kaduna State, Nigeria.

**FIGURE 4:**
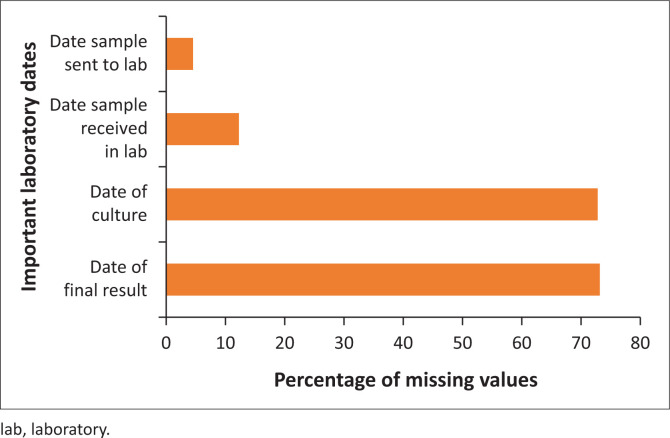
Proportion of missing values for laboratory data in the 2023 outbreak line-list in Kaduna State, Nigeria.

Eleven (52%) of the 21 respondents rated the clarity of data collection as ‘Good’, while 10 (48%) of the 21 respondents rated the care taken in completing the surveillance forms equally as ‘Good’. Twelve (57%) of the 21 respondents reported that relevant authorities have supervised them in the past. A review of the outbreak line-list for variables with missing values showed that individuals’ date of birth had 382 (67%) missing values out of 573 individuals (Figure 3). The proportion of missing values for laboratory data were 352 (73%) out of the 483 laboratory samples collected (Figure 4).

#### Acceptability

Twenty-one (100%) respondents reported a willingness to continue to participate in the system, although 11 (52%) admitted that they have problems with carrying out their responsibilities effectively. Twenty-one (100%) respondents reported that the system appreciated them. Twelve (57%) of the 21 respondents agreed that they have made suggestions to improve the system and 8 (67%) of those 12 respondents noted that their recommendations were implemented.

#### Sensitivity

Twenty-one (100%) respondents reported that the surveillance system could correctly identify those who have diphtheria and that there have been no misdiagnosed cases in their respective Local Government Areas. Nineteen (90%) reported that there were no missing cases who had tested positive, while 17 (81%) noted that they were satisfied with the outcomes of the laboratory tests. However, a key informant cited that the ‘laboratory turnaround time needs improvement’.

#### Representativeness

Twenty-one (100%) respondents reported that data tools used in diphtheria surveillance depicted all the necessary information on distribution of cases. Nineteen (90%) of the 21 respondents stated that all public health facilities in their respective Local Government Areas provided their reports, while 12 (57%) of the 21 respondents stated the same for private health facilities.

#### Timeliness and completeness

Results from the interviews show that Kaduna State has 31 reporting focal sites. The timeliness and completeness in comparison with the WHO standards^3^ are shown in Table 1.

**TABLE 1:** Timeliness and completeness in comparison to the World Health Organization standards.

Surveillance attribute	Indicator	Target (%)	Calculation	Observed value (%)
Completeness of reporting	Percentage of designated sites reporting diphtheria data, even in the absence of cases (zero reporting)	≥ 80	Total number of reports received/total number of reporting sites × 100 (for given time period) (31/31*100)	100
Timeliness of reporting	Percentage of Local Government Areas (LGAs) reporting to the State level on time, even in the absence of cases	≥ 80	Number of LGAs in the state reporting by the deadline/Number of LGAs in the state × 100 (23/23*100)	100
Timeliness of investigation	Percentage of all suspected diphtheria cases that have had an investigation initiated within 48 h of notification.	≥ 80	Number of suspected cases of diphtheria for which an investigation initiated (samples collected) within 48 h of notification/Number of suspected diphtheria cases × 100 (432/525*100)	82

#### Stability

Twelve (56%) of the 21 respondents reported that they do not have stipends to carry out their work as DSNOs. Out of the nine respondents who reported stipends, six (67%) said the stipends were from partner organisations involved in diphtheria surveillance. In addition, 19 (90%) of the 21 respondents reported that they do not have adequate resources for surveillance activities.

## Discussion

The diphtheria surveillance system in Kaduna State met most of the qualities of a good surveillance system with gaps in timely outbreak detection, data quality and representativeness. The evaluation’s strength lies in the willingness of the stakeholders to participate in the assessment and continue in the system.

The system may be considered useful to stakeholders; however, there was a delay in detecting the index case of the 2023 outbreak, which may have occurred as early as Epi week 18. Such delay has implications for timely response and may hinder the system’s effectiveness in preventing and controlling outbreaks.^20,21,22^ The system functions simply, with most respondents reporting the usage of straightforward forms for data collection that were completed with ease. These findings are similar to those from a diphtheria surveillance system evaluation carried out in Indonesia. However, unlike this study, the researchers also evaluated the simplicity of logistics management for diphtheria antitoxin (DAT) and sample collection, and found them not to be simple.^23^

The system has shown some degree of flexibility to change, as nearly half of the participants reported that modifications were quickly incorporated into the data-gathering routine which was corroborated by a key informant. This finding aligns with a similar study in Delhi, India, to evaluate the measles and diphtheria surveillance system which was adjudged to be flexible in adjusting to variations over time.^24^ The results on data quality suggest that the clarity of data collection and the completion of surveillance forms were generally evaluated as ‘Good’, indicating an average performance. However, the high percentages of missing values in the line-list suggest possible problems with the accuracy and completeness of the data. This can undermine evidence-based decision-making within the system.^25^ To address this, it may be helpful to introduce standardised and advanced data-capturing techniques,^25^ including the use of technologies.^26^ Furthermore, the frequency of supervision sessions was found to be below ideal levels,^11^ which could negatively impact the prompt identification and resolution of data quality issues. Findings are similar to that of the measles and diphtheria evaluation in Delhi, India, where data quality was good for case data, but was poor for mortality data.^24^

Despite difficulties in fulfilling their duties efficiently, respondents are quite willing to stay involved in the system. The occurrence of difficulties like inadequate incentives emphasises how crucial it is to address major issues to increase motivation and involvement.^27^ These findings contrast with a similar study carried out in East Java, Indonesia, to evaluate the diphtheria surveillance system which was found to have low acceptability.^28^

The results indicate that the system is sensitive, because all respondents attested to the system’s capacity to correctly identify cases. These findings are in consonance with the study in Delhi, India, to evaluate the measles and diphtheria surveillance system, where the system was sensitive in detecting trends, although it lacks population data to estimate disease burden.^24^ The findings are, however, in contrast with an evaluation carried out in East Java, Indonesia, where the system was reported to have low sensitivity.^28^

The findings on the representativeness of the system suggest that, even while it captures pertinent aspects of the disease, there might be gaps in the data provided by private healthcare facilities. Most respondents reported receipt of data from public health facilities compared to private facilities. This aligns with the findings from the study in Delhi, India, that showed paucity of data reported from private facilities.^24^ A malaria surveillance system evaluation done in Kano state, Nigeria also gave similar result of poor surveillance data from private facilities.^29^

A high degree of timeliness in both reporting and investigation is evident from the results. Most suspected cases were investigated within 48 h of notice, and all focal sites routinely provided their weekly reports on schedule. The timeliness of reporting and investigation was, respectively, above the recommended WHO target.^3^ Further research is, however, needed to know why the 2023 outbreak was not detected timely as revealed from analysis of the outbreak data. Further research may also be required to explore other aspects of timeliness in the surveillance system. The completeness of reporting is demonstrated by the fact that all reporting sites consistently provided their reports, including zero reporting. The completeness of reporting was also above the recommended WHO target.^3^ Further research may also be needed to explore other aspects of completeness in the system and to explore the inclusion and improvement of sites to capture private health facilities in the state.

Findings from this evaluation raise concerns regarding the stability of the surveillance system. The absence of specialised and sufficient surveillance resources threatens both the reliability and the availability of the system. The fact that external partner groups provide stipends emphasises the system’s additional susceptibility to changes in external funding. The evaluation of the malaria surveillance system in Kano state, Nigeria, similarly revealed a donor-dependent^29^ system. To guarantee any surveillance system’s long-term stability and functionality, resource deficiencies must be filled.^27,30^

For the limitation of this evaluation, the survey relied on self-reported data from respondents, which may introduce response bias or inaccuracies. The sample size of respondents may not fully represent all perspectives of the entire surveillance system, potentially limiting the generalisability of the findings. Finally, the evaluation did not explore the perspectives of key stakeholders such as community members who could provide valuable insights into broader system dynamics.

## Conclusion

The diphtheria surveillance system in Kaduna State is useful in detecting diphtheria cases but has gaps in timely outbreak detection. While the system demonstrates simplicity and effectiveness in data collection and reporting, there are concerns about the poor reporting from private health facilities. Additionally, the system showed some degree of flexibility; however, resource constraints pose a threat to its stability.

Data quality issues highlight the need for improved supervision and training. Despite challenges, stakeholders remain willing to participate in the system, indicating strong acceptability. The system’s sensitivity in identifying cases is commendable, but efforts are needed to improve timely outbreak detection.

### What is already known about this topic?

- Diphtheria continues to be endemic in several countries, especially those with sub-optimal diphtheria vaccination coverage.
- Public health surveillance is relevant for prompt identification and response to public health threats and outbreaks.
- The diphtheria surveillance system is aimed at monitoring diphtheria burden and defining transmission patterns; identifying outbreaks promptly to avert spread; and full implementation of national policy on diphtheria vaccination.

### What this study adds?

- Diphtheria is an immediately case-based notifiable disease in Nigeria and the surveillance in Kaduna State is in accordance with Nigeria’s National Technical Guidelines for IDSR system.
- One of the states affected by the 2023 outbreak of diphtheria in Nigeria is Kaduna State. The re-emergence of diphtheria outbreak in Nigeria started in May 2023, and as of October 2023, Kaduna State was reported to be one of the six worst-hit states, contributing to over 95% of cases and 600 deaths.
- The re-emergence of diphtheria outbreak in Nigeria raises several public health concerns, including issues bordering on the capacity of the surveillance system to promptly detect, prepare and respond to the outbreaks.
